# Glutamate levels across deep brain structures in patients with a psychotic disorder and its relation to cognitive functioning

**DOI:** 10.1177/02698811221077199

**Published:** 2022-03-04

**Authors:** Tommy AA Broeders, Alex A Bhogal, Lisan M Morsinkhof, Menno M Schoonheim, Christian H Röder, Mirte Edens, Dennis WJ Klomp, Jannie P Wijnen, Christiaan H Vinkers

**Affiliations:** 1 Department of Radiology, University Medical Center Utrecht, Utrecht, The Netherlands; 2 Department of Anatomy & Neurosciences, Amsterdam Neuroscience, Amsterdam UMC, Vrije Universiteit Amsterdam, Amsterdam, The Netherlands; 3 Department of Psychiatry, Brain Center Rudolf Magnus, University Medical Center Utrecht, Utrecht, The Netherlands; 4 Department of Psychiatry, Amsterdam Neuroscience, Amsterdam UMC, Vrije Universiteit Amsterdam/GGZ inGeest, Amsterdam, The Netherlands

**Keywords:** Magnetic resonance spectroscopic imaging (MRSI), proton spectroscopy, glutamate, psychotic disorder, cognition, psychomotor speed

## Abstract

**Background:**

Patients with psychotic disorders often show prominent cognitive impairment. Glutamate seems to play a prominent role, but its role in deep gray matter (DGM) regions is unclear.

**Aims:**

To evaluate glutamate levels within deep gray matter structures in patients with a psychotic disorder in relation to cognitive functioning, using advanced spectroscopic acquisition, reconstruction, and post-processing techniques.

**Methods:**

A 7-Tesla magnetic resonance imaging scanner combined with a lipid suppression coil and subject-specific water suppression pulses was used to acquire high-resolution magnetic resonance spectroscopic imaging data. Tissue fraction correction and registration to a standard brain were performed for group comparison in specifically delineated DGM regions. The brief assessment of cognition in schizophrenia was used to evaluate cognitive status.

**Results:**

Average glutamate levels across DGM structures (i.e. caudate, pallidum, putamen, and thalamus) in mostly medicated patients with a psychotic disorder (*n* = 16, age = 33, 4 females) were lower compared to healthy controls (*n* = 23, age = 24, 7 females; *p* = 0.005, *d* = 1.06). Stratified analyses showed lower glutamate levels in the caudate (*p* = 0.046, *d* = 0.76) and putamen *p* = 0.013, *d* = 0.94). These findings were largely explained by age differences between groups. DGM glutamate levels were positively correlated with psychomotor speed (*r*(30) = 0.49, *p* = 0.028), but not with other cognitive domains.

**Conclusions:**

We find reduced glutamate levels across DGM structures including the caudate and putamen in patients with a psychotic disorder that are linked to psychomotor speed. Despite limitations concerning age differences, these results underscore the potential role of detailed in vivo glutamate assessments to understand cognitive deficits in psychotic disorders.

## Introduction

Patients with psychotic disorders often show prominent cognitive deficits that greatly impact their quality of life, and these deficits determine long-term outcomes more than positive symptoms (i.e. hallucinations and delusions) (Kahn and Keefe, 2013; McCleery and Nuechterlein, 2019). These cognitive deficits often precede psychosis onset and persist after the acute phase of the illness and despite pharmacological treatment (McCleery and Nuechterlein, 2019; Reichenberg et al., 2010; Woodberry et al., 2008). However, the underlying pathophysiology of cognitive deficits in psychotic disorders is currently poorly understood. Glutamatergic dysfunction is involved in the development and course of psychotic symptoms, including cognitive deficits (Bojesen et al., 2020, 2021; Egerton et al., 2018; Howes et al., 2015; Merritt et al., 2013; Theberge et al., 2002; Wenneberg et al., 2020), and assessing glutamate levels may be important for understanding these cognitive deficits (Bojesen et al., 2020, 2021; Bustillo et al., 2011; Egerton et al., 2018; Howes et al., 2015; Merritt et al., 2013; Theberge et al., 2002; Wenneberg et al., 2020).

The main technique for directly assessing in vivo glutamate levels is proton magnetic resonance spectroscopy (^1^H-MRS) (Wijtenburg et al., 2015). The majority of ^1^H-MRS studies have focused on frontal or medial cortical areas (Marsman et al., 2013; Poels et al., 2014), but recently there has been increased attention on glutamatergic disturbances in subcortical regions like the thalamus and striatum where glutamatergic deficits may be pronounced (Bojesen et al., 2020, 2021; de la Fuente-Sandoval et al., 2013; Egerton et al., 2018; Kim et al., 2018; Marsman et al., 2013; Plitman et al., 2019; Poels et al., 2014; Reddy-Thootkur et al., 2020). These regions are thought to play a key role in the pathophysiology and etiology of psychosis (Avery et al., 2021; Chan et al., 2021; Gangadin et al., 2021; Grace, 2016). On top of that, these structures play a prominent role in cognitive functioning (Beste et al., 2018; Bicanski and Burgess, 2020; Wolff et al., 2021), and glutamate levels in these subcortical regions may be particularly important for understanding cognition in schizophrenia, but also disease progression in individuals at clinical high risk (Allen et al., 2015; Avery et al., 2021; Bossong et al., 2019).

Deep brain structures are interesting targets for ^1^H-MRS(I) studies seeking to quantify suspected alterations in glutamate and glutamine (and their combined signal (Glx)) levels in patients with psychotic disorders. Nevertheless, reported changes have been mixed. A recent meta-analysis, summarizing ^1^H-MRS studies at 1.5–4 Tesla in schizophrenia, reported elevated Glx (Merritt et al., 2016), while earlier meta-analyses reported more heterogeneous results (Marsman et al., 2013; Poels et al., 2014). This variability may at least partially arise from limited signal-to-noise ratios and effects relating to overlapping resonances in the glutamate levels at lower magnetic field strength when using single-voxel sequences, including the separation of glutamate and glutamine resonances. Moreover, MRS studies typically involve the acquisition of a limited number of single voxels obtained unilaterally. These voxels of interest have volumes between 5 and 8 cm^3^ that lead to significant mixing of gray matter (GM) and white matter (WM) signals—that is, reducing sensitivity to changes in GM due to partial volume effects with WM. With these considerations in mind, the experimental design can be limited with respect to *which* deep brain structure can/should be targeted when investigating disease-mediated neurochemical changes and their relation to cognitive deficits. Magnetic resonance spectroscopic imaging (MRSI) at ultra-high field (⩾7 Tesla) can circumvent these limitations due to increased spectral dispersion and higher field-strength-dependent signal-to-noise ratio (SNR). Increased spectral dispersion facilitates a better distinction of Glu from potentially overlapping resonances, while increased SNR provides the currency to acquire high-resolution metabolic images with reduced partial volume effects that can intersect several brain structures simultaneously.

The aim of this study was therefore to acquire MRSI data of deep brain glutamate levels in patients with a psychotic disorder and examine its relation to cognitive deficits. To allow the simultaneous targeting of multiple deep brain regions in a single acquisition, we used a high-resolution two-dimensional (2D) MRSI approach at 7 Tesla. Novel methodological combinations were applied to enable group analyses on glutamate levels across delineated deep brain regions. In line with previous studies investigating deep brain regions post-treatment, we hypothesized that patients with a psychotic disorder would show reduced glutamate values in the striatum and thalamus, which is associated with cognitive dysfunction.

### Materials and methods

#### General

Subjects were recruited at the University Medical Center Utrecht (UMCU). Inclusion criterion for patients was the presence of a psychotic disorder as assessed using the Mini-International Neuropsychiatric Interview (MINI) (Sheehan et al., 1998). Patients with a drug-induced psychosis or an affective disorder with psychotic features as assessed with the MINI (Sheehan et al., 1998) were excluded. Individuals without lifetime neurological or psychiatric conditions were recruited as healthy controls, and metabolite maps of these participants have been previously published (Bhogal et al., 2020). All participants were requested to abstain from recreational drugs in the 2-week period prior to the study and from alcohol within 24 h prior to the study. This study was approved by the Medical Research Ethics Committee of UMCU, and written informed consent was obtained from all subjects and was performed according to the guidelines and regulations of the WMO (Wet Medisch Wetenschappelijk Onderzoek) and the Declaration of Helsinki.

#### Psychiatric evaluation and medication

The MINI was administered before study participation by graduate students who were trained by an experienced psychiatrist. Study participants were asked to list the names and doses of any drugs they were taking for psychosis. The reported daily doses were divided by the assumed average maintenance dose (i.e. the defined daily dose (DDD)), as reported by the World Health Organization (http://www.whocc.no/) (Leucht et al., 2016).

#### Cognitive assessment

The Brief Assessment of Cognition in Schizophrenia (BACS) was used for the evaluation of cognitive status and administered before magnetic resonance imaging (MRI) scanning on the same day (Keefe et al., 2004). The BACS included eight items covering six domains: verbal memory, working memory, motor speed, verbal fluency (three items), attention and processing speed, and executive function. The raw scores of each item were transformed to *z-*scores based on the distribution of the healthy controls, by subtracting the mean of that item and dividing by the standard deviation of control values for that item. The three items for verbal fluency were averaged, resulting in six *z*-scores per participant, indicating the cognitive score per domain. Finally, all six z-scores were averaged per participant, and average scores were used as a basis to quantify cognition.

#### MRI data

**Figure 1.**
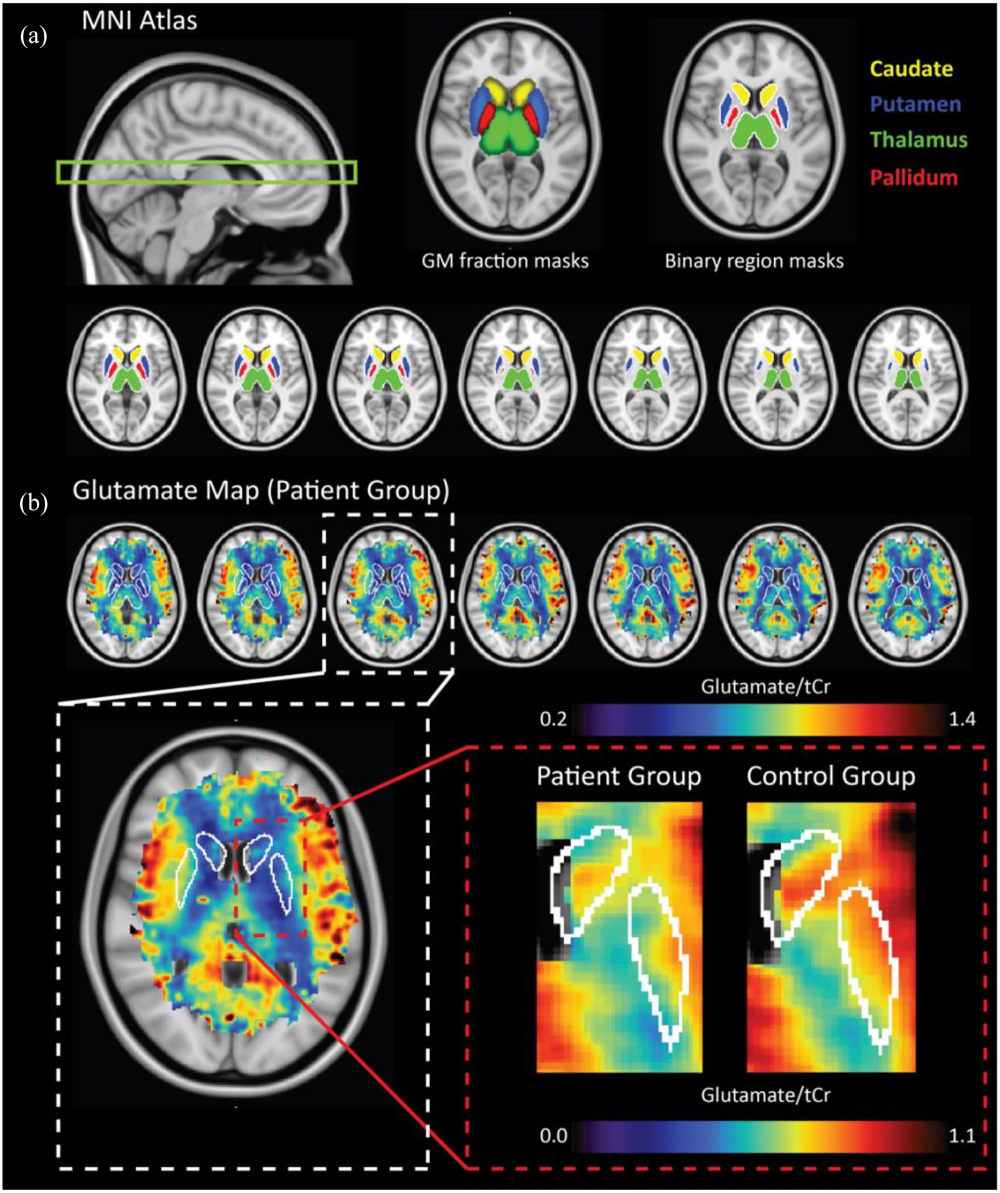
(a) Deep GM regions were delineated based on tissue masks associated with the MNI standard atlas. GM tissue fraction maps (middle) were thresholded to create binary masks (right) of the caudate (>30%), putamen (0%), thalamus (>60%), and pallidum (>10% GM). The target region for our acquisition is shown in the sagittal image (left). Transverse anatomical MNI images along with corresponding average Glu maps for the patient group are shown (right); (b) top: selected transverse slices of the average Glu map from the patient group are shown along with delineated ROI; bottom: zoomed images showing average data between patients (left) and controls (right) for the caudate and putamen, where significant reductions in Glu were observed. Figure scaling has been adjusted to highlight localized differences.

Data were acquired using a 7-Tesla MR scanner (Phillips, Best, NL, USA) equipped with a dual-transmit head coil and 32-channel proton receive coil (Nova Medical, Wilmington, MA, USA). Second-order image-based shimming was as achieved using an external lipid suppression coil (Boer et al., 2015). MRSI data were acquired using a slice-selective, free-induction decay sequence (Bogner et al., 2012) with the following parameters: echo time (TE)/repetition time (TR) = 2.5/300 ms, field of view (FOV) = 220 × 220 mm, acquisition matrix = 44 × 44, voxel size = 5 × 5 × 10 mm^3^, bandwidth (BW) = 3000 Hz, samples = 512, signal averages = 2, elliptical k-space, scan duration = 10 min 59 s, subject-specific spiral in-out spectral-spatial water suppression pulses (Ma et al., 2018), flip angle = 35°. Water-unsuppressed MRSI data were acquired for zeroth-order phase and eddy current correction with adapted parameters: acquisition matrix = 22 × 22, resolution = 10 × 10 × 10 mm^3^, signal averages = 1, scan duration = 1 min 54 s. Two adjacent 10 mm MRSI slices (20 mm slab) were acquired axially to intersect deep GM nuclei located adjacent to the ventricles (Figure 1(a)). This region was selected based on previous indications of glutamatergic dysfunction related to psychiatric disorder. Finally, a metabolite-nulled spectrum was obtained in a single subject in order to acquire a macromolecular (MM) baseline signal (Bhogal et al., 2020).

A three-dimensional Turbo Field Echo (TFE) scan (TE/TR = 2.89/8 ms, resolution = 1 mm isotropic, FOV = 220 × 220 × 200 mm^3^, scan duration = 6 min 51 s, flip angle = 6°), a shimmed dual-echo GRE B_0_ map (delta TE/TR = 2.35/5.12 ms, FOV = 220 × 220 × 30 mm^3^, acquisition matrix = 176 × 176, resolution = 1.25 × 1.25 × 10 mm^3^, slices = 3, scan duration = 1 min 6 s), and 2D multi-slice T1w Fast Field Echo (FFE) image (TE/TR = 4.22/200 ms, FOV = 220 × 220 × 30 mm^3^, acquisition matrix = 176 × 176, resolution = 1.25 × 1.25 × 10 mm^3^, slices = 3, scan duration = 72 s) were acquired.

#### Spectral fitting and quantification

The MRSI data reconstruction steps are fully described in the work by Bhogal et al. (2020) which outlines the generation of the healthy reference values used for comparison in this study. The same approach was followed to generate the patient metabolite date reported in this work. The FFE and B_0_ map were used to perform over-discrete reconstruction with B_0_ correction (Kirchner et al., 2017) or spectral data, while the whole-brain TFE anatomical image facilitated spatial normalization of MRSI data to the 1-mm MNI atlas. Spectra were fit using LCModel (Provencher, 2001). The basis set included GPC (glycerophosphocholine), Cho (choline), phosphocholine, tCr (creatine and phosphocreatine), Glu (glutamate), Gln (glutamine), taurine, myo-inositol, glycine, glucose, NAA (N-acetyl-aspartate), NAAG (N-acetyl-aspartyl-glutamate), gamma-aminobutyrate, aspartate, glutathione, lactate, succinate, guanidoacetate, scyllo-inositol, acetate, and the measured macromolecular baseline signal. Lipid resonances were simulated by LCModel. Spectra having an SNR lower than 3, full width at half maximum (FWHM) higher than 0.15, Cramér–Rao Lower Bound (CRLB) (of Glu or tCR) higher than 50%, or lipid-to-creatine (Lip13a/tCr) ratio >2 were not included for further analysis (Bhogal et al., 2020). Due to the B_0_ distortions of the external lipid suppression coil, a majority of these voxels were located at the periphery of the cerebrum. Remaining voxels were used to generate 2D maps of glutamate. Tissue segmentations (GM, WM and cerebrospinal fluid (CSF)) were used to perform high-resolution partial volume and T1 correction of the Glu maps. Here, CSF contributions were considered negligible, while the remaining Glu signals were re-distributed based on pure tissue values. Not only did this approach provide more accurate delineation of deep GM (DGM) structures, but it also takes into consideration that the broad point-spread-function was associated with the low-resolution MRSI acquisition matrix (for a complete description, see Bhogal et al. (2020) and the accompanying Supplementary Data).

Region-of-interest masks of brain regions from which sufficient coverage was attained (i.e. the caudate, putamen, thalamus, and pallidum) were generated using MNI tissue fraction maps (Figure 1(a)) provided in FSL (FMRIB) (Smith et al., 2004). The tissue fraction masks were binarized by including only voxels exceeding specific %GM as outlined in Figure 1. Region-specific thresholds were chosen so as to minimize WM contributions while maintaining as much as GM tissue as possible. Left and right regions were combined into a single mask. The combined deep GM region was created by amalgamating these regions of interest into a single mask (Figure 1(b)). The average glutamate levels were computed for each mask if signal from at least 10% of the entire nuclei was attained; below that coverage was regarded too little to reliably quantify glutamate levels of that region. However, to verify that the effects were not dependent on this arbitrary threshold, pair-wise comparisons were computed with varying thresholds from 5% to 35% with 5% increments (for details, see Supplementary Materials).

#### Statistical analyses

To compare demographic characteristics, independent *t*-tests and chi-square tests were performed. The average glutamate values in the combined deep GM region were compared between patients and controls using a general linear model with glutamate values as dependent variable and group as independent variable. Stratified analyses were subsequently performed on individual deep GM regions to assess whether any effects could be further localized to individual regions; these analyses were of an exploratory nature and no correction for multiple comparisons across regions was applied. Sensitivity analyses were performed for all group comparisons, by performing the analyses again using age as covariate. For brain regions that differed between patients and controls, Spearman’s correlations between glutamate levels and potentially confounding variables were calculated, that is, age and the use of drugs for psychosis, and *t*-tests were performed to assess differences between sexes and patients who are or are not currently treated with drugs for psychosis or drugs for anxiety. The relation between glutamate levels and overall cognition as well as individual cognitive domains was assessed using Spearman’s correlations corrected for group-effects (and age-effects in an additional sensitivity analysis) and applying false discovery rate correction to account for multiple comparisons across cognitive domains. Statistical analyses were performed in SPSS (version 26.0.0.1). We checked normality using histogram inspection, and *p*-values less than 0.05 were considered statistically significant. All results are reported as means ± standard deviation.

## Results

### Participant characteristics

Demographics and clinical characteristics are displayed in Table 1. In total, 23 healthy control subjects were recruited from the general population (age 24 ± 6 years, 9 females) and 16 individuals with a psychotic disorder (age 33 ± 11 years, 4 females). The average age of patients was higher (*p* = 0.008), but gender ratios were comparable (*p* = 0.711). Eight patients were diagnosed with schizophrenia, six with psychosis not otherwise specified, and two with a schizoaffective disorder. As expected, overall BACS scores were lower in patients with a psychotic disorder compared to controls (*t*(37) = 3.36, *p* = 0.002). For the individual cognitive domains, scores were significantly lower for verbal memory (*p* = 0.012) and attention/processing speed (*p* < 0.001), but not for working memory, psychomotor speed, verbal fluency, and executive functioning (all *p*-values >0.05).

**Table 1.** Demographics and clinical parameters.

	Controls (*N* = 23)	Patients (*N* = 16)	Pairwise comparisons	
			Test statistic	*p*-value
Age	23.87 (±5.7)	33.13 (±11.7)	*t*(20) = −2.93	0.008*
Gender (F/M)	7/16	4/13	χ^2^(38) = 0.14	0.711
Average cognition	0.19 (±0.66)	−0.76 (±0.81)	*t*(37) =−3.36	0.002*
Verbal memory	0.04 (±1.01)	−1.50 (±2.06)	*t*(20) = 2.76	0.012*
Working memory	0.06 (±1.04)	−0.47 (±1.34)	*t*(37) = 1.40	0.196
Psychomotor speed	0.06 (±1.04)	−0.56 (±1.12)	*t*(37) = 1.779	0.083
Verbal fluency	0.01 (±0.88)	−0.37 (±0.72)	*t*(37) = 1.432	0.161
Attention/processing speed	0.02 (±1.00)	−1.40 (±1.24)	*t*(37) = 3.949	<0.001*
Executive functioning	0.00 (±1.00)	−0.26 (±0.79)	*t*(37) = 0.882	0.383
Drugs				
For psychosis (Y/N)	–	13/3		
Defined daily dose (*n* = 6)	–	0.76 (±0.41)		
For depression (Y/N)	–	4/12		
For relapse prevention (Y/N)	–	2/14		
Drugs for anxiety (Y/N)	–	5/11		
Diagnosis (SZ, PN)	–	8/6/2		
Age of onset psychosis	–	23.67 (±10.1)		
Years since first psychosis	–	10.71 (±11.5)		

F: female; M: male; Y: yes; N: no; SZ: schizophrenia; PN: psychosis not otherwise specified; SA: schizoaffective disorder.

* *p* < .05.

### Analysis of possible confounders

In both patients and controls, age was significantly related to glutamate levels in the combined deep GM (*r*(32) = −0.43, *p* = 0.010), but no differences in glutamate values were found between men and women (*t*(32) = 0.57, *p* = 0.571). Within patients only, the DDD of the drugs for psychosis was not significantly correlated to deep GM glutamate levels (*r*(5) = −0.38, *p* = 0.40), neither were there differences between patients who did or did not take drugs for psychosis (*t*(12) = 0.00, *p* = 0.999) or drugs for anxiety (*t*(10.253) = 1.04, *p* = 0.322), but these results were based on a small sample, and the lack of a significant relationship should be interpreted with caution.

### Glutamate levels in deep brain structures

**Figure 2.**
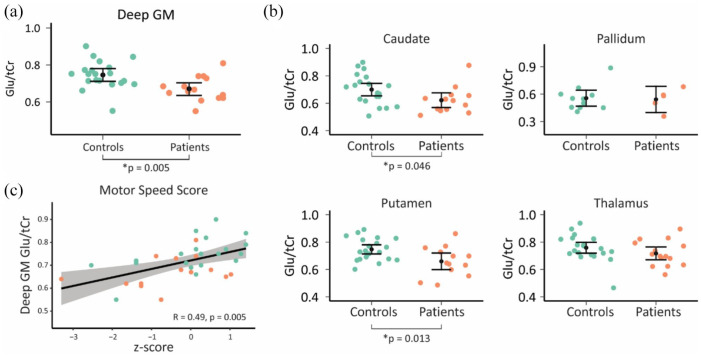
The Glu/tCr ratio found per subject in the control and patient groups. (a) The ratios were portrayed for the deep gray matter (DGM) as a whole. (b) Glutamate levels per anatomically defined brain region with sufficient data to perform statistical analysis. Means and 95% confidence intervals per group are superimposed on the individual data points. Significant differences were found in whole deep GM, and the caudate and putamen (*p* < 0.05). (c) Deep GM glutamate levels positively correlate to psychomotor speed (Spearman’s correlation, adjusted for age and group). The motor speed score represents a normalized value relative to the healthy control values (i.e. *z*-scores). Glu: glutamate; tCr: total creatine; GM: gray matter.

Compared to healthy controls, overall glutamate levels in combined deep GM structures were significantly lower in patients with a psychotic disorder compared to healthy controls (0.67 ± 0.07 vs 0.75 ± 0.08; *F*(1, 32) = 8.88, *p* = 0.005, *d* = 1.06) (Figure 2(a)). Stratified analyses showed significantly lower glutamate levels in the caudate (0.62 ± 0.10 vs 0.70 ± 0.11; *F*(1, 31) = 4.32, *p* = 0.046, *d* = 0.76) and putamen (0.66 ± 0.11 vs 0.75 ± 0.08; *F*(1, 31) = 6.93, *p* = 0.013, *d* = 0.94), but not in the pallidum (0.59 ± 0.13 vs 0.52 ± 0.09; *F*(1, 14) = 0.04, *p* = 0.853, *d* = 0.15) or thalamus (0.72 ± 0.09 vs 0.76 ± 0.10; *F*(1, 34) = 1.66, *p* = 0.206, *d* = 0.42; Figure 2(b)). Sensitivity analyses with different coverage signal thresholds (5–35%) showed that decreased deep GM glutamate levels were present across coverage signal thresholds, even though the statistical power due to decreasing sample size affected statistical significance to a certain degree (see Supplementary Figure S1). A representative spectrum showing the data quality and the associated LCModel fit is provided in Supplementary Figure 2. Including age as a covariate removed significant group differences (deep GM: *p* = 0.070; caudate: *p* = 0.272; putamen: *p* = 0.126).

### Glutamate levels in relation to cognition

Overall, cognitive scores were not significantly correlated to glutamate values across deep GM structures (*r*(31) = 0.30, *p* = 0.086; age-adjusted: *r*(30) = 0.26, *p* = 0.154). Subsequently, we analyzed individual cognitive domain scores in relation to combined deep GM glutamate levels. A significant relation was found between glutamate levels and psychomotor speed (*r*(31) = 0.48, *p* = 0.031; age-adjusted: *r*(30) = 0.49, *p_corr_* = 0.028; Figure 2(c)), but not for verbal memory (*r*(31) = 0.13, *p* = 0.715; age-adjusted: *r*(30) = 0.11, *p_corr_* = 0.590), working memory (*r*(31) = −0.02, *p* = 0.912; age-adjusted: *r*(30) = −0.12, *p_corr_* = 0.590), verbal fluency (*r*(31) = 0.10, *p* = 0.715; age-adjusted: *r*(30) = 0.15, *p_corr_* = 0.590), attention/processing speed (*r*(31) = 0.22, *p* = 0.631; age-adjusted: *r*(30) = 0.15, *p_corr_* = 0.590), and executive functioning (*r*(31) = 0.11, *p* = 0.715; age-adjusted: *r*(30) = 0.10, *p_corr_* = 0.590). Stratified analyses did not show statistically significant correlations for psychomotor speed in the caudate (*r*(30) = 0.13, *p* = 0.478; age-adjusted: *r*(29) = 0.14, *p* = 0.442), putamen (*r*(30) = 0.25, *p* = 0.166; age-adjusted: *r*(29) = 0.25, *p* = 0.171), thalamus (*r*(33) = 0.28, *p* = 0.100; age-adjusted: *r*(32) = 0.29, *p* = 0.100), or pallidum (*r*(11) = −0.18, *p* = 0.555; age-adjusted: *r*(10) = −0.18, *p* = 0.574).

## Discussion

Using advanced 2D MRSI techniques, this study found reduced glutamate levels in deep GM structures in patients with a psychotic disorder, which was particularly pronounced in the caudate and putamen. However, age differences between groups are a complicating factor and preclude definitive evidence of these results. We have found that deep GM glutamate positively related to psychomotor functioning and not to verbal memory, working memory, verbal fluency, attention and processing speed, and executive function. Our approach included a high-resolution 7-Tesla acquisition with precise delineation of deep GM structures while mitigating sources of error through novel artifact control techniques and correction for tissue-specific GM and WM glutamate contributions. This is the first 7-Tesla study to report the quantification of glutamate across anatomically delineated deep GM regions simultaneously in patients with a psychotic disorder, in contrast to single-voxel spectroscopic measurements.

In support of our findings, studies employing higher magnetic field strengths have generally reported reduced glutamate levels in schizophrenia patients (Dempster et al., 2020; Kumar et al., 2020; Marsman et al., 2014; Reid et al., 2019; Thakkar et al., 2017; Wang et al., 2019), even though these studies generally focused on cortical areas (anterior cingulate cortex or occipital cortex) rather than deep brain structures. Another previous study at 3 Tesla did focus on the striatum, however, and reported increased glutamate prior to drug treatment for psychosis which normalized post-treatment (de la Fuente-Sandoval et al., 2013). Several cellular mechanisms are possible through which glutamate levels can be altered in psychotic disorders. Blocking the N-methyl-d-aspartate (NMDA) receptor through ketamine administration in healthy humans has shown to increase glutamate (Stone et al., 2012) and glutaminase (Ye et al., 2013), the key enzyme to convert glutamine to glutamate, although these findings were not corroborated by all studies (Bojesen et al., 2018; Taylor et al., 2012). Changes in the NMDA receptor or glutaminase activity might cause a shifting glutamate/glutamine balance (Marsman et al., 2013). Studies investigating specifically intra- and extrasynaptic glutamate levels are needed to understand the relationship between glutamate concentration and NMDA receptor functioning, which, unfortunately, is not possible using MRSI. Our finding of overall reduced glutamate levels contrasts with a recent meta-analysis finding of elevated Glx signal in patients with schizophrenia (Merritt et al., 2016), but very few studies have quantified bilateral glutamate levels across deep brain structures. Moreover, it is important to note that MRSI studies performed at lower magnetic field strength may be hindered by lower SNRs, greater partial volume effects due to large voxel size, and more ambiguities in signal quantification due to overlapping glutamate and glutamine resonances.

Cognitive deficits have a pronounced effect on the quality of life and functional outcome of patients with a psychotic disorder (Kahn and Keefe, 2013; McCleery and Nuechterlein, 2019). We found that glutamate levels in deep brain regions are involved in psychomotor outcomes, and not cognitive functioning in general. These results complement the findings from a previous 4 Tesla MRSI study (Bustillo et al., 2011), as that study found that cortical glutamate levels positively related to a range of cognitive domains in schizophrenia patients. Although overall cognition was impaired in our patient sample, psychomotor speed was not. Nevertheless, psychomotor speed problems are a well-known phenomenon in patients who have experienced a psychosis (van Harten et al., 2017). Deep GM regions, including striatal structures (e.g. putamen and caudate), are involved in motor actions(Florio et al., 2018). Moreover, previous research has shown that the putamen was involved in psychomotor speed (Shirinbayan et al., 2019). Thus, these findings suggest that altered glutamate levels in deep brain regions could relate to altered psychomotor functioning, whereas glutamate levels in cortical regions might be more strongly related to other cognitive processes. Furthermore, a recent study suggested a link between structural disruptions of the striatum and negative symptoms in schizophrenia patients (Cobia et al., 2021), so future studies are suggested to investigate a possible link between striatal glutamate and (negative) symptom severity as well.

Notwithstanding important methodological improvements, our study has several limitations. First, the sample is limited in size and consisted of patients who have taken drugs for psychosis with a range of disease durations. This is important as these drugs may influence glutamate levels (Bojesen et al., 2020; de la Fuente-Sandoval et al., 2013; Egerton et al., 2018) and have effects on psychomotor speed and may even exacerbate cognitive problems through dysregulation of NMDA receptor activity (Ballesteros et al., 2018). Similarly, a better understanding is needed on whether glutamate levels in deep GM structures are decreased already prior to treatment and whether the same regions display glutamate level alterations before and after treatment. Second, in order to remove extra-cranial lipid signal contamination, we explicitly focused on deep brain structures to improve the accuracy of metabolite quantification, but this comes at the cost of cortical glutamate signals for which analyses were not possible due to the use of extra-cranial signal suppression. Similarly, the limited spatial coverage of our acquisition meant that central areas lower in the brain were not fully acquired or may have been contaminated due to susceptibility artifacts common to those regions. This may explain the rather sparse data available in the pallidum region for patients (see Figure 2).

Correcting for the effects of age removed significant group differences in glutamate levels, and group differences in age were large, so this limits interpretation. Our study can be regarded as a pilot study since replication of these findings in well-sized, age-matched samples with clearly documented medication use and dose or drug-naïve patients is needed. Furthermore, studies investigating glutamate levels at earlier stages could elucidate whether glutamate plays an important role in the development of psychomotor deficits. Finally, creatine was used as internal reference, in line with current literature (Posse et al., 2013), to divide out deviations related to coil loadings and inhomogeneous B1 transmit/receive fields. A few studies have suggested that creatine levels might be reduced in schizophrenia patients, so this effect might mask the differences in glutamate levels between patients and controls (Merritt et al., 2021; Ongur et al., 2009). This further emphasizes the salience of the glutamate reduction that was observed in this study.

As technological innovations expand the applicability of MRSI as a method to evaluate neuronal metabolism, future studies will benefit from emerging techniques for whole-brain metabolic imaging. This necessity is underscored by our observation that glutamate levels in deep GM structures primarily related to psychomotor functioning, while cortical glutamate, as measured by Bustillo et al. (2011), related to broader cognitive domains. A whole-brain approach would make it possible to localize specific metabolic consequences and relate them to associated known functional domains simultaneously to generate cognitive profiles. By further accelerating spectroscopic imaging techniques at high resolutions, functional magnetic resonance spectroscopy can also provide insights into the dynamic aspects of neuronal metabolism that may also be altered due to psychiatric disease (Stanley and Raz, 2018).

### Conclusion

Our results highlight the value of applying high-resolution MRSI to understand cognitive and motor deficits related to psychotic disorders by quantifying glutamate levels across anatomically defined subcortical brain structures. Moreover, our study shows that novel methodological methods to measure detailed levels of in vivo glutamate across deep brain structures are important tools to understand the mechanisms of psychotic disorders.

## Supplemental Material

sj-docx-1-jop-10.1177_02698811221077199 – Supplemental material for Glutamate levels across deep brain structures in patients with a psychotic disorder and its relation to cognitive functioning Click here for additional data file. Supplemental material, sj-docx-1-jop-10.1177_02698811221077199 for Glutamate levels across deep brain structures in patients with a psychotic disorder and its relation to cognitive functioning by Tommy AA Broeders, Alex A Bhogal, Lisan M Morsinkhof, Menno M Schoonheim, Christian H Röder, Mirte Edens, Dennis WJ Klomp, Jannie P Wijnen and Christiaan H Vinkers in Journal of Psychopharmacology
