# Supplementary material for: Glutamate levels across deep brain structures in patients with a psychotic disorder and its relation to cognitive functioning
Source: J Psychopharmacol. 2022 Mar 4;36(4):489–97. doi: 10.1177/02698811221077199 (PMC9066676; doi:10.1177/02698811221077199)
Supplement: sj-docx-1-jop-10.1177_02698811221077199 – Supplemental material for Glutamate levels across deep brain structures in patients with a psychotic disorder and its relation to cognitive functioning [file sj-docx-1-jop-10.1177_02698811221077199.docx]

**SUPPLEMENTARY INFORMATION**


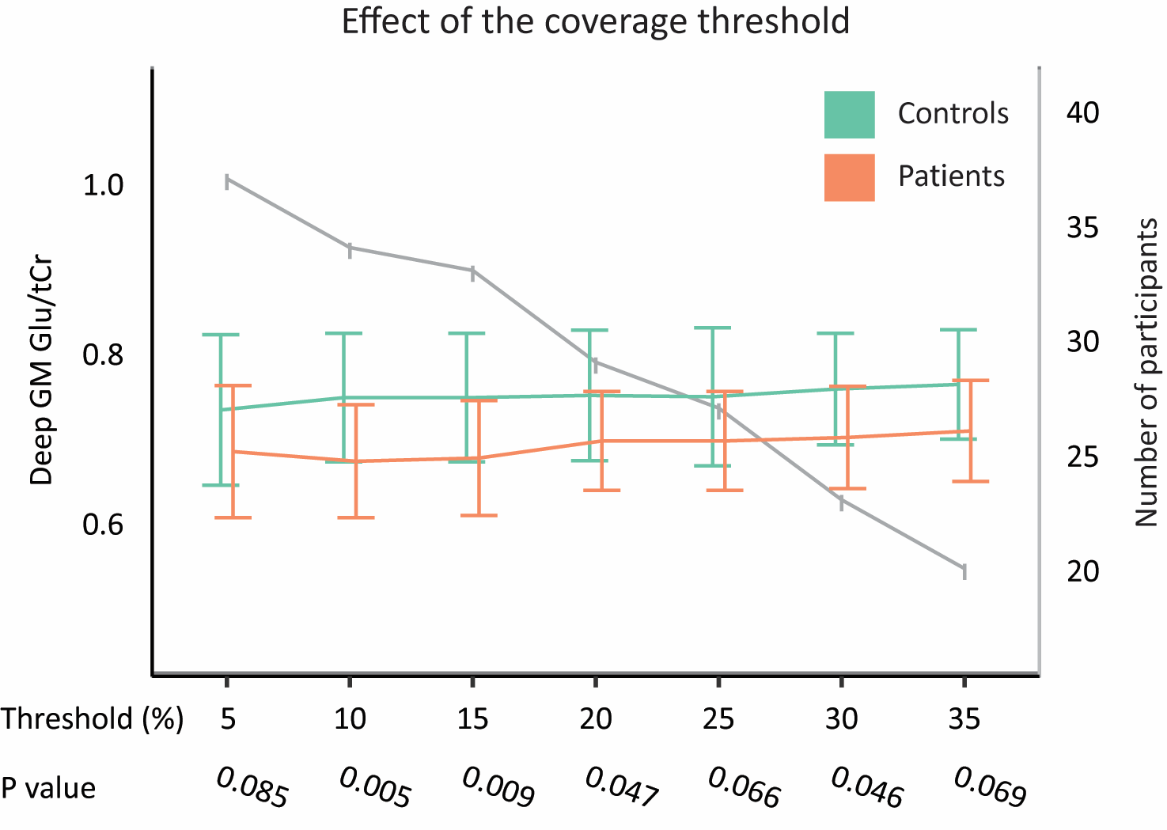


**Supplementary Figure S1.** Sensitivity analyses with a range of coverage (i.e. the percentage of deep brain structure covered by the MRS-signal) signal thresholds showed that decreased deep GM glutamate levels were present across coverage signal thresholds, even though the statistical power due to decreasing sample size affected statistical significance to a certain degree. With increasing threshold, the variance of the two groups was reduced as more coverage would lead to a more reliable signal and reduce the possibility of outliers. The x-axis represents the coverage threshold. The blue and green lines represent the Glu/tCr values in patients (blue) and controls (green; left y-axis), at the same time the grey line shows the number of patients remaining after thresholding (right y-axis). The means were calculated for every 5% increment and plotted with slight offsets around the threshold for better visualization, the error bars represent the standard deviations. Glu = Glutamate, tCr = Total Creatine, GM = gray matter.
